# Dense is not green: How visual density influences greenness evaluation on environmentally friendly products

**DOI:** 10.3389/fpsyg.2022.1035021

**Published:** 2023-01-09

**Authors:** Chunqu Xiao, Haoyuan Wang, Yayu Zhou, Qingyi Li

**Affiliations:** ^1^College of Business, Shaoxing University, Shaoxing, China; ^2^School of Management, University at Buffalo, Buffalo, NY, United States; ^3^School of Business, Nanjing University, Nanjing, China

**Keywords:** greenness evaluation, environmentally friendly products, visual density, holistic thinking, metaphorical meaning

## Abstract

**Introduction:**

The visual design of environmentally friendly products has a strong influence on consumer decisions. The study offers a novel insight, suggesting that consumers' perceptions of environmentally friendly products may be affected by the visual density design.

**Methods:**

Four experiments tested the effect of visual density on the perceived greenness of environmentally friendly products.

**Results:**

Study 1 showed that perceived greenness was higher for environmentally friendly products with low visual density design. Study 2 repeatedly confirmed this impact and found that perceived production cost acted as a mediating factor. Study 3 and 4 found two boundary conditions for this effect. Study 3 showed that the effect of visual density design attenuated for consumers with weak holistic thinking. Study 4 further revealed that when emphasizing the use of environment-friendly materials, the effect of visual density design was also attenuated.

**Discussion:**

The findings enrich the discussion on the visual design of green products, extend the effect of visual density on consumer attitudes, and provide practical implications for marketers to choose the appropriate appearance for environmentally friendly products.

## 1. Introduction

Beneficial to our world, enhancing environmental awareness plays a critical role in ecological protection (Hu et al., 2021; Zhou et al., 2021; Chen and Wu, 2022; Khan et al., 2022; Maitlo et al., 2022). Such a trend brings pro-environment and green consumption as important selling points in marketing (Shen and Wang, 2022). At present, many marketers label their products as “environmentally friendly” or “green” to establish a responsible brand image and attract consumers (Gomez-Banderas, 2022). With the increasing popularity of green consumption, the study of green consumption occupies an important place in the research on consumer psychology (Khan et al., 2022). Existing research explored how green attributes (Gershoff and Frels, 2015), hedonistic value (Olsen et al., 2012), durability (Sun et al., 2021), symbol value (Chan et al., 2012), and perceived quality (Ariffin et al., 2016) influence consumers' greenness evaluation or purchase intention on environmentally friendly products.

However, prior research that centered on the visual design of environmentally friendly products was relatively limited in scope. Advertising research on confirmed that the visual factor plays an essential role in the publicity of environmental protection (Spack et al., 2012; Pancer et al., 2017; Zhang et al., 2019). However, the effects of visual factors on environmentally friendly products have still not been given enough attention. In particular, numerous research works on visual designs that affect greenness evaluation are needed. This study aimed to fill the gap by demonstrating that green-minded consumers realize that high visual density design does not necessarily mean being sustainable and environmentally friendly.

Consumers think that environmentally friendly products cause less pollution and require fewer resources (Luchs et al., 2010; Delmas and Burbano, 2011). Conversely, high visual density designs may imply using many materials and resources. Such a link comes from the symbolic metaphor of visual features. As a newly proposed visual feature, researchers recently pointed out that the pattern design with high visual density makes consumers feel “filled” (Su et al., 2019). The current research suggests that the “filled” feeling refers to the latent meaning of “plenty” of resources, which leads to a conflict between high visual density and environmental protection concepts. As a consequence, consumers believe that a low visual density design is more beneficial to the environment than a high visual density design, enhancing their propensity to buy low visual-density designed environmentally friendly items.

This study explores the impact of the visual density of environmental protection products on consumer evaluation. It enriches prior research in several points. First, it fills the lack of research on the visual characteristics of environmental protection products. Second, it also extends the effect of visual density. Third, it adds to the work on the metaphor of visual features. It provides support for the contextual feature of metaphorical meaning. In practical implications, this study offers a feasible strategy for marketers to raise consumers' perceptions of environmentally friendly products and instructs marketers to select the appropriate visual design for such products.

## 2. Theoretical review

### 2.1. Environmentally friendly product

Compared with conventional products, green products refer to products with at least one positive environmentally friendly attribute (Testa et al., 2021). Based on this definition, a product may qualify as environmentally friendly if it possesses only one green feature. Studies about consumers' evaluation and purchase intention on environmentally friendly products belong to the field of green consumption. Green consumption refers to the consumption behavior characterized by saving resources and protecting the environment (Luchs et al., 2010; Delmas and Burbano, 2011; Haws et al., 2014). The existing research on green consumption involves two aspects. One aspect of green consumption research explores how individual factors influence consumers' green consumption based on psychodynamic theories (e.g., theory of planned behavior, normative activation theory, and self-determination theory) (Zepeda and Deal, 2009; White et al., 2019; Ruangkanjanases et al., 2020). The internal factors include personal values (Sharma and Batra, 2015; Sreen et al., 2018), personal ability factors (e.g., green knowledge and perceived effectiveness) (Yadav and Pathak, 2017; Arli et al., 2018), and behavior factors (habits and practices) (Cervellon et al., 2012; Kumar et al., 2017). For instance, Wells et al. (2010) confirmed that women are more open to green consumption. Martenson (2017) found that consumers with high self-awareness are more likely to buy green products. Sreen et al. (2018) reported that collectivism is significantly related to green purchase intention. Kumar et al. (2017) found that past purchases of recyclable packaging will increase the willingness to purchase similar products in the future.

The second aspect of green consumption research reveals the impact of external factors on green consumption behavior (Testa et al., 2021). The majority of the external elements are situational factors, such as product green content, advertising, and social norms (Spack et al., 2012; Gershoff and Frels, 2015; Wu et al., 2015; Pancer et al., 2017; He and Zhan, 2018; Trivedi et al., 2018; Zhang et al., 2019). Compared with individual factors, the research on external factors is relatively limited. Specifically, the attention bestowed to the appearance design of green products is insufficient. Existing research primarily focused on the color design of environmentally friendly products or environmental protection advertisements (ads) (Spack et al., 2012; Pancer et al., 2017; Zhang et al., 2019). Prior research found that green color could improve consumers' greenness evaluation of environmentally friendly products (Spack et al., 2012). Zhang et al. (2019) found that people pay more attention and give more positive ratings to blue ads about protecting the sea or green ads about protecting the forest. The current research suggests the effect of visual density on environmental protection products, aiming to enrich the discussion on the appearance design of green products.

### 2.2. Visual density

Visual density refers to the number of visual elements in the unit area of visual design (Donderi, 2006; Rosenholtz et al., 2007). In physics, density is defined as the mass per unit volume or the degree of being dense (Su et al., 2019). Density can also be described as the compactness of elements per unit. For example, temporal density or “busy” indicates a situation where many tasks need to be done in a short period (Snyder, 2013). This study focuses on visual density, which is an important visual feature for products (Su et al., 2019). Current studies investigated how visual features, such as color, irregularity, symmetry, and design style, affect the judgment and decision of consumers (Hagtvedt and Patrick, 2008; Deng and Kahn, 2009; Hoegg and Alba, 2011; Hagtvedt and Brasel, 2017). However, discussion about the research on visual density has been held only to a limited extent.

It is of great significance for researchers and marketers to explore how visual density influences consumers' evaluation and understand the underlying explanation mechanism of this effect. For instance, a website with a high-density design has many easily recognizable features (Su et al., 2019). Previous research underlined that low visual density decreases the difficulty in information processing and that low visual density patterns are associated with psychological emptiness (Pieters et al., 2010; Su et al., 2019). In keeping with the research trend, researchers pursuing current research seek to find out how visual density design influences consumers' greenness perception of environmentally friendly products.

## 3. Research hypotheses

As was established, green consumption refers to consumption behavior characterized by saving resources and protecting the environment (Testa et al., 2021). Environmentally friendly products consume fewer resources to reduce environmental pressure (Luchs et al., 2010; Delmas and Burbano, 2011; Gershoff and Frels, 2015). Consumers believe that the products with “Environmentally friendly” or “Green” labels should consume fewer resources and require lower production costs than those without such labels (Luchs et al., 2010; Delmas and Burbano, 2011; Gershoff and Frels, 2015). The definition of visual density in conceptual structure is analogous to the production cost per product. To be precise, the former refers to the number of manufacturing resources used to produce a unit of a product, while the latter refers to the number of visual elements contained in a unit area. The schema theory predicts that concepts with similar structures interact and assimilate each other in the cognition of consumers (Schmidt, 1975). Different concepts are interrelated accordingly, leading to metaphorical links between sensory input and abstract concepts (Landau et al., 2010; Krishna and Schwarz, 2014). For example, social rank and physical spatial hierarchy show the structure of “high” to “low.” For this reason, people link social status with spatial hierarchy in their cognition and agree that “higher” in spatial structures implies “higher” social status (Schwartz et al., 1982; Fiske, 2004). For another example, most people have the habit of reading from left to right. “Left” and “Right” are two words used to indicate orientations. Under such circumstances, the direction of flowing from left to right is formed. Time also has a direction to flow. The research found that left and right in space have a metaphorical connection with the past and future in the sense of time (Boroditsky, 2000). In short, the structural consistency of concepts provides the possibility for metaphor.

There is evidence to suggest that visual design density has symbolic significance. Previous research confirmed that low-density visual design is related to the feeling of emptiness (Su et al., 2019). This is due to the fact that lower visual density leads to fewer elements in the pattern, leaving a large blank and making consumers associate such emptiness with psychological emptiness, for example, the lack of regular peer group contact (Su et al., 2019). While a pattern with a high visual density is packed with numerous visual components, it may cause consumers to feel full. The dense pattern containing more visual elements can be metaphorically linked to the psychological perception of being filled, which indicates belonging to a coherent community and feeling more social connections (Su et al., 2019).

Similarly, this study suggests that visual density may also be metaphorically linked to the perceived production cost of products. Visual density refers to the number of visual elements in the unit area of visual design (Donderi, 2006; Rosenholtz et al., 2007), and production costs refer to all the costs from manufacturing a product or the amount of resource consumption per product. Following prior research on metaphorical links, we assume that consumers can link the number of visual elements in the esthetic design to the amount of product resource consumption. There will be a significant blank on the package pattern when the product packaging was designed with low visual density, suggesting the inclusion of fewer visual elements, which gives consumers the impression that the product was produced with fewer resources. This fact is consistent with the core characteristics of green products in saving resources. The study accordingly believes that lower visual density helps to improve the greenness evaluation of consumers on green products. We formally state the following hypotheses.

H1: Low (vs. high) visual density design can improve the perceived greenness of environmentally friendly products.

H2: This effect is mediated by the perceived production cost of products.

### 3.1. Moderation effect of consumers' holistic thinking

The effect of low visual density design to improve the greenness evaluation of environmentally friendly products requires consumers to pay attention to the structural similarity between the number of resources contained in the unit product and the concept of visual density, which links the density of visual elements in the design with the consumption of product resources. For those consumers who do not tend to notice the similarity between concepts, the effect of visual density design on the evaluation of green products will be mitigated. Consumers who notice the similarity between concepts can more easily link visual density design to the greenness evaluation of environmentally friendly products. Such a personal trait that tends to notice the similarity between concepts is called consumers' holistic thinking (Burns and Shepp, 1988; Orth and Malkewitz, 2008; Hildebrand et al., 2019). People with stronger holistic thinking tendencies are more likely to see the similarities between concepts/things and are more inclined to connect these concepts/things with similar characteristics. However, those people with a weak holistic mindset pay more attention to the differences between concepts and the independent characteristics of things. If consumers link visual density design to greenness evaluation through the path of metaphor, the effect of visual density on greenness evaluation will be attenuated for consumers with a weak holistic mindset. Thus, testing the moderation effect of holistic thinking can help support the metaphorical connection between visual density and perceived production cost. We formally state the following hypothesis.

H3: The effect of visual density on greenness evaluation is mitigated for consumers with a weak holistic thinking tendency.

### 3.2. Moderation effect of emphasizing the use of environment-friendly materials

High visual density can decrease the evaluation of green products because consumers associate the number of visual elements in dense patterns with resource/materials' cost. Prior research showed that when changing the objects/concepts associated with visual stimulus, the link between visual stimulus and objects/concepts will change accordingly (Meier et al., 2012; Zhang and Han, 2014). For example, when the color blue is associated with clean water, consumers will respond positively to the blue stimulus (Palmer and Schloss, 2010); but when blue is associated with meat, consumers will view the blue stimulus unfavorably (Eckstut and Eckstut, 2013). We assume that if consumers associate visual density design with environment-friendly materials, the negative influence of high visual density design, which means the use of more environment-friendly materials then, will be mitigated.

In addition to the consumption of fewer resources, sustainability is another important attribute of green products (Luchs et al., 2010; Sun et al., 2021). This attribute requires green products should use more recyclable and environment-friendly materials. For example, some bags are made of automatically degradable starch. If consumers were reminded of the use of environment-friendly materials, for example, by presenting relevant descriptions, they could link dense patterns with using more environment-friendly materials. Then, the negative effect of dense design on greenness evaluation may be mitigated. Using fewer resources, whether environment-friendly materials or not, meets the requirements of environmental protection (Donderi, 2006; Rosenholtz et al., 2007). Therefore, we did not assume the effect of visual density on greenness evaluation in reverse when presenting the description of using environment-friendly materials. We formally state the following hypothesis.

H4: The effect of visual density design on the greenness evaluation of environmentally friendly products is mitigated when emphasizing the use of environment-friendly materials.

## 4. Experiment 1: The influence of visual design density on consumers' green product evaluations

The main purpose of Experiment 1 was to test the effect of visual design density on consumers' green product evaluation (H1). We preregistered this study (https://aspredicted.org/GMS_XY1).

### 4.1. Design and participants

Experiment 1 used a between-subjects design with visual design density (high vs. low). Two hundred two participants from the United States of America were recruited from Amazon's Mechanical Turk (MTurk). We used Qualtrics, a professional online survey platform (www.qualtrics.com). As preregistered, participants who failed to pass the color blindness test were excluded, leaving the final sample size as 193 participants (62.7% women, *M*_age_ = 42.42 ± 1.64 years).

### 4.2. Procedure

After being informed about the purpose of the research and study procedures, participants were randomly assigned either to high visual design density situations or to low visual design density situations. They were presented with an image of tissue either with a high visual density pattern or with a low visual density pattern (see Appendix A). The patterns were taken from prior research on visual density (Su et al., 2019). Participants were told the product is “tissue with sustainability certifications.” Then, participants were told to evaluate the perceived greenness of the tissue using two items (adapted from Gershoff and Frels (2015): “this tissue is a good environmental choice,” “this tissue is environmentally friendly”; 1 = “strongly disagree” and 7 = “strongly agree”; *r* = 0.85). The average score constituted the dependent variable. Last, participants reported their age and gender.

### 4.3. Results

#### 4.3.1. Perceived greenness

**Figure 1:**
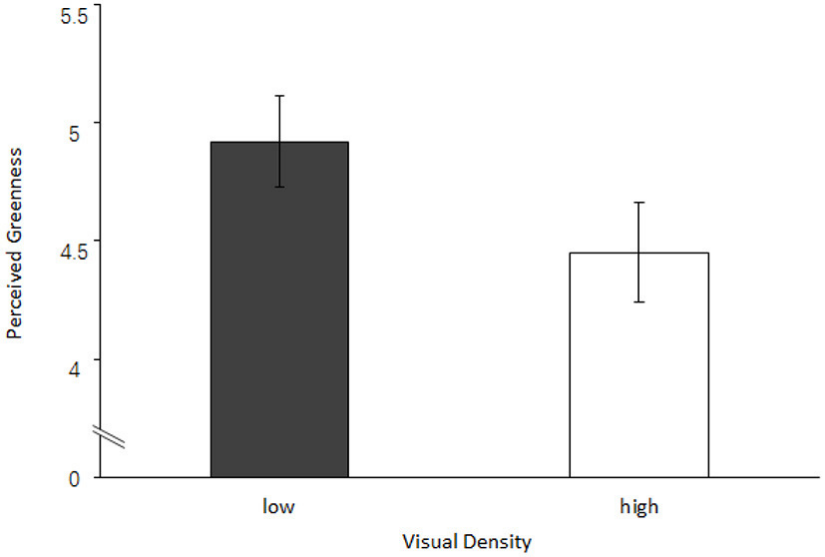
The effect of visual density on perceived greenness.

The t-test was used to examine the effect of visual design density on perceived greenness. We found that participants' perceived greenness in the high visual density group was significantly lower (*M*_low_ = 4.92, SD = 1.37, *M*_high_ = 4.45, SD = 1.50; *t*_(105)_ = −2.26, *p* = 0.025, Cohen's d = −0.32) (Figure 1). Therefore, Experiment 1 shows that visual design density has a significant impact on consumers' green product evaluation.

### 4.4. Discussion

Experiment 1 preliminarily verified the main effect that consumers' perceived greenness was higher for environmental protection products with low (vs. high) visual design density. The results provided support for H1.

## 5. Experiment 2: Mediating effect of perceived production cost

Experiment 2 changed the materials of visual design density to replicate the main effect and improve the credibility of the research results. Experiment 2 also explored the inner mechanism of the effect of visual design density on consumers' green product evaluation. Prior research found that complex visual design could increase emotional arousal and decrease consumers' long-term preferences (Buechel and Townsend, 2018). Since sustainability is another important attribute of green products (Luchs et al., 2010; Sun et al., 2021) and consumers expect that environmentally friendly products should be durable (Sun et al., 2021), we measured consumers' arousal level as an alternative explanation in this experiment.

### 5.1. Design and participants

Experiment 2 used a between-subjects design with visual design density (high vs. low). One hundred twenty participants were recruited from online social communities of a University in Nanjing. Participants completed the tasks online in exchange for a small payment. We used Sojump, a professional survey platform (https://www.sojump.com) used by prior research (Liu et al., 2021) to record their responses. Two participants failed to pass the color blindness test, leaving the final sample with 118 participants (59.3% women, *M*_age_ = 23.83 ± 0.73 years).

### 5.2. Procedure

After being informed about the purpose of research and study procedures, participants were randomly assigned either to high visual design density situations or to low visual design density situations. Participants saw an image of a phone case with a high visual density pattern or a low visual density pattern (see Appendix A). Also, the patterns were taken from prior research on visual density (Su et al., 2019). Participants were told that the product is “a phone case with sustainability certifications.” Then, participants were told to evaluate the perceived greenness using the same two items that were used in Study 1 (*r* = 0.88) and the perceived production cost on three items (“the merchant saves a lot of resources when making the mobile phone case,” “the merchant saves a lot of time when making the mobile phone case,” and “the merchant saves a lot of energy when making the mobile phone case”; 1 = “strongly disagree” and 7 = “strongly agree”; Cronbach's α = 0.90) adopted from Fuchs et al. (2015). Then, participants were told to report their purchase intention on three items (“I am willing to buy this product for environmental protection,” “I am willing to use this product for environmental protection,” and “I am willing to search for relevant information of this product for environmental protection”; 1 = “strongly disagree” and 7 = “strongly agree”; Cronbach's α = 0.93) as a measurement of the downstream effect. After that, participants' arousal level [from Bradley and Lang (1994): 1 = “This product is sluggish/calm/relaxed” and 9 = “This product is frenzied/excited/stimulated”] was also assessed. Last, participants reported their age and gender.

### 5.3. Results

#### 5.3.1. Perceived greenness

The t-test was used to examine the effect of visual design density on perceived greenness. The results revealed that participants' perceived greenness in a high (vs. low) visual density group was significantly lower (*M*_low_ = 4.71, SD = 1.40, *M*_high_= 4.05, SD = 1.49; *t*_(116)_ = −2.47, *p* = 0.015, Cohen's d = −0.46).

#### 5.3.2. Perceived production cost

**Figure 2:**
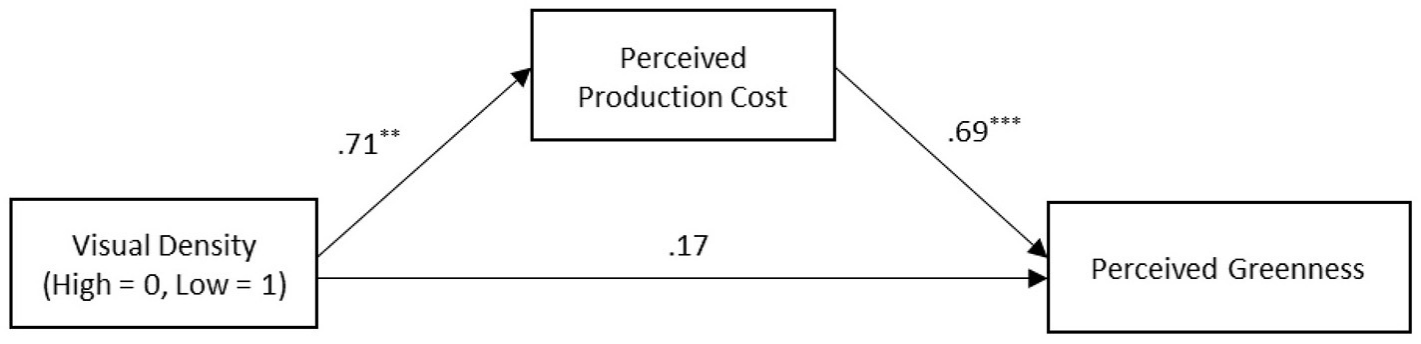
The mediating effect of perceived production cost. *p <0.1, **p <0.05, and***p <0.01. Unstandardized coefficients are reported.

Visual design density significantly predicted perceived production cost. The product with low visual density was perceived to save more resources in production (*M*_low_ = 5.02, SD = 1.42, *M*_high_= 4.31, SD = 1.20; *t*_(116)_ = −2.94, *p* = 0.004, Cohen's d = −0.55). To test the mediating effect of perceived production cost, a bootstrapping analysis with 5,000 resamples (PROCESS Model 4; Hayes, 2017) was conducted (Figure 2). The results revealed that visual density (high = 0, low = 1) significantly predicted perceived production cost (*b* = 0.71, SE = 0.24, CI_95%_ [.23, 1.19]) and perceived production cost significantly predicted perceived greenness (*b* = 0.69, SE = 0.08, CI_95%_ [0.53, 0.84]). The indirect effect was significant (*b* = 0.49, SE = 0.19, CI_95%_ [0.15, 0.90]). The direct effect was not significant (*b* = 0.17, SE = 0.22, CI_95%_ [−0.26, 0.60]).

#### 5.3.3. Arousal

The *t-*test was used to examine the effect of visual design density on arousal. The results showed that the effect of visual design density on arousal was not significant (*M*_low_ = 3.53, SD = 2.14, *M*_high_= 3.93, SD = 1.93; *t*_(116)_ = 1.09, *p* = 0.278). When including arousal and perceived production cost in the bootstrapping analysis (PROCESS Model 4; Hayes, 2017) as mediators at the same time, we found that the indirect effect of perceived production cost was significant (*b* = 0.46, SE = 0.19, CI_95%_ [0.12, 0.85]), but the indirect effect of arousal was not significant (*b* = −0.04, SE = 0.04, CI_95%_ [−0.14, 0.03]). Taken together, arousal cannot explain the effect of visual density on greenness evaluation.

#### 5.3.4. Purchase intention

The *t-*test was used to examine the effect of visual design density on purchase intention. The results revealed that participants' purchase intention for environmentally friendly products with a low (vs. high) visual density design was higher (*M*_low_ = 4.71, SD = 1.37, *M*_high_ = 4.06, SD = 1.64; *t*_(116)_ = −2.36, *p* = 0.020, Cohen's d = −0.44). A bootstrapping analysis with 5,000 resamples (PROCESS Model 4; Hayes, 2017) was conducted with visual density as the independent variable, purchase intention as the dependent variable, and perceived production cost as the mediator. The results revealed that visual density (high = 0, low = 1) significantly predicted perceived production cost (*b* = 0.71, SE = 0.24, CI_95%_ [0.23, 1.19]) and perceived production cost significantly predicted purchase intention (*b* = 0.63, SE = 0.09, CI_95%_ [0.45, 0.81]). The indirect effect was significant (*b* = 0.45, SE = 0.19, CI_95%_ [0.14, 0.87]). The direct effect was not significant (*b* = 0.20, SE = 0.24, CI_95%_ [−0.27, 0.68]).

### 5.4. Discussion

Experiment 2 used different experimental materials of visual design density, verified the main effect again, and proved the mediating effect of perceived production cost. The results supported H2. In addition, Experiment 2 proved that arousal could not explain the effect of visual density. We also tested the downstream consequences of this effect and found that low visual design density could improve consumers' purchase intention of environmentally friendly products. Experiments 3 and 4 will explore further the boundary conditions that could influence the effect of visual design density.

## 6. Experiment 3: The moderation effect of consumers' holistic thinking tendency

Experiment 3 tested the moderation effect of consumers' holistic thinking tendency. We added measurement on holistic thinking and expected that the effect of visual density design on greenness evaluation is mitigated for consumers with a weak holistic thinking tendency.

### 6.1. Design and participants

Experiment 3 used a between-subjects design with visual design density (high vs. low). One hundred twenty-five participants who did not participate in Study 2 were recruited from online social communities of a University in Nanjing. Participants completed the tasks online in exchange for a small payment. We used Sojump, a professional survey platform (https://www.sojump.com) used by prior research (Liu et al., 2021) to record their responses. Five participants failed to pass the color blindness test, leaving the final sample size as 120 participants (51.7% female, M_age_ = 24.63 ± 0.74 years).

### 6.2. Procedure

After being informed about the purpose of research and study procedures, participants were randomly assigned either to a situation with high visual design density or to a situation with low visual design density. As in Experiment 1 and Experiment 2, participants saw an image of a *T-*shirt with a high visual density pattern or a low visual density pattern (see Appendix A) and the patterns were taken from prior research on visual density (Su et al., 2019). Participants were told the product is “an environmentally-friendly *T-*shirt.” Participants were told to evaluate the perceived greenness on two items used in Study 1 (*r* = 0.86). Then, we measured participants' holistic thinking tendency by taking five items from prior research (Choi et al., 2003): “Even a small change in any element in the universe can lead to substantial alterations in others,” “Any phenomenon has a numerous number of results although some of the results are not known,” “The whole is greater than the sum of its parts,” “A marker of good architecture is how harmoniously it blends with other buildings around it,” and “Sometimes, the empty space in a painting is just as important as the objects”; 1 = “strongly disagree” and 9 = “strongly agree”; Cronbach's α = 0.87). Then, participants reported their age and gender.

### 6.3. Results

#### 6.3.1. Perceived greenness

The *t-*test was used to examine the effect of visual design density on perceived greenness. The results revealed that participants' perceived greenness in a high (vs. low) visual density group was significantly lower (*M*_low_ = 5.38, SD = 1.40, *M*_high_ = 4.76, SD = 1.73; *t*_(118)_ = −2.17, *p* = 0.032, Cohen's d = −0.40).

#### 6.3.2. Moderation effect of holistic thinking

To test the moderation effect of holistic thinking, a bootstrapping analysis with 5,000 resamples (PROCESS Model 1; Hayes, 2017) using visual design density as the independent variable, perceived greenness as the dependent variable, and holistic thinking as the moderator was conducted. The results revealed that the interaction between visual density and holistic thinking was significant (*b* = 0.43, SE = 0.19, CI_95%_ [0.05, 0.82]). The main effect of visual density was non-significant (*b* = −2.65, SE = 1.41, CI_95%_ [−5.44, 0.15]), and the main effect of holistic thinking was non-significant (*b* = −0.06, SE = 0.28, CI_95%_ [−0.49, 0.62]). Further spotlight analyses revealed that low visual density increased perceived greenness for participants with a mean score (M) (*b* = 0.46, SE = 0.24, CI_95%_ [0.01, 0.93]) and a relatively high score (M + 1 SD) (*b* = 1.02, SE = 0.34, CI_95%_ [0.35, 1.69]) on holistic thinking, but the effect of visual density was mitigated for participants with a relatively low holistic thinking score (M-1 SD) (*b* = −0.09, SE = 0.35, CI_95%_ [−0.78, 0.59]) (Table 1).

**Table 1:** The moderation effect of holistic thinking.

**Holistic thinking score**	**Effects of visual design density on Perceived greenness**		
	***b***	**SE**	**CI_95%_**
M – 1SD	−0.09	0.35	[−0.78, 0.59]
M	0.46	0.24	[0.01, 0.93]
M + 1SD	1.02	0.34	[0.35, 1.69]

### 6.4. Discussion

Experiment 3 replicated the effect of visual design density on perceived greenness and further found that, for participants with relatively low holistic thinking scores, the effectof visual density was mitigated, showing the moderation effect of holistic thinking (H3). The results showed that, for consumers who do not tend to find the link between different concepts, low (vs. high) visual density design could not improve the perceived greenness of environmentally friendly products, revealing the metaphorical connection between visual density and perceived production cost in this effect.

## 7. Experiment 4: The moderation effect of emphasizing the use of environment-friendly materials

Experiment 4 tested the moderation effect of emphasizing the use of environment-friendly materials. We expected that, when emphasizing the use of environment-friendly materials, consumers associate visual density with environment-friendly materials' use. The high visual density design implies using more environment-friendly materials, which may not improve the perceived wastage of resources and energy.

### 7.1. Design and participants

Experiment 4 used a 2 (visual design density: high vs. low) ×2 (emphasis on the use of environment-friendly materials: with vs. without) between-subjects design with 226 participants who did not participate in Study 2 and Study 3 recruited from online social communities of a University in Nanjing. Participants completed the tasks online in exchange for a small payment. We used Sojump, a professional survey platform (https://www.sojump.com) used by prior research (Liu et al., 2021), to record their responses. Four participants failed to pass the color blindness test, leaving the final sample with 222 participants (50.9% female, M_age_ = 22.41 ± 0.38 years).

### 7.2. Procedure

After being informed about the purpose of research and study procedures, participants were randomly assigned either to high visual design density situations or to low visual design density situations. As in Experiment 1 and Experiment 2, participants saw an image of a coffee cup with a high visual density pattern or a low visual density pattern (see Appendix A). The patterns were taken from prior research on visual density (Su et al., 2019). Participants were told the product is “an environmentally-friendly coffee cup” or “an environmentally-friendly coffee cup made from coffee grounds and paper.” We presented coffee grounds here as an environment-friendly material. Then, participants were told to evaluate the perceived greenness on the same two items used in Study 1 (*r* = 0.64) and the perceived production cost on three items used in Study 2 (Cronbach's α = 0.83). Last, participants reported their age and gender.

### 7.3. Results

#### 7.3.1. Perceived greenness

**Figure 3:**
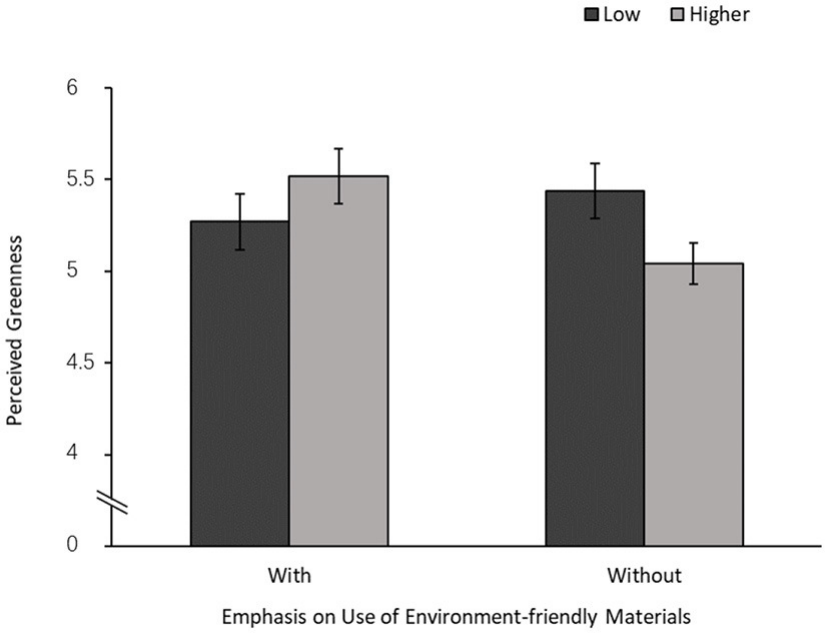
The moderation effect of emphasis on the use of environment-friendly materials.

A 2 × 2 analysis of variance (ANOVA) was conducted on perceived greenness (Figure 2). The interaction between visual design density and emphasis on the use of environment-friendly materials was significant [*F*_(1,218)_ = 5.65, *p* = 0.018, partial η^2^ = 0.03] (Figure 3). The main effects of visual design density [*F*_(1,218)_ = 0.29, *p* = 0.589] and emphasis on the use of environment-friendly materials [*F*_(1,218)_ = 1.32, *p* = 0.252] were not significant.

Further examination of the interaction revealed that the effect of visual design density was not significant when the use of environment-friendly materials was emphasized [*M*_low_ = 5.27, SD = 1.07, M_high_ = 5.52, SD = 1.10; *F*_(1,218)_ = 1.71], but low visual density could improve perceived greenness without such emphasis [M_low_ = 5.44, SD = 0.80, M_high_ = 5.04, SD = 1.07; *F*_(1,218)_ = 4.18, *p* = 0.042, partial η^2^ = 0.02].

#### 7.3.2. Perceived production cost

A 2 × 2 analysis of variance (ANOVA) was conducted on perceived production cost. It showed a significant interaction between visual design density and emphasis on the use of environment-friendly materials [*F*_(1,218)_ = 5.55, *p* = 0.019, partial η^2^ = 0.025]. The main effects of visual design density [*F*_(1,218)_ = 0.21, *p* = 0.650] and emphasis on the use of environment-friendly materials [*F*_(1,218)_ = 0.50, *p* = 0.481] were not significant.

Further examination of the interaction revealed that the effect of visual design density on perceived production cost was not significant when the use of environment-friendly materials was emphasized [M_low_ = 4.71, SD = 1.15, M_high_ = 5.01, SD = 1.15; *F*_(1,218)_ = 1.84, *p* = 0.176], but the product with low visual density was perceived to save more resources in production without such emphasis [M_low_ = 5.19, SD = 1.06, M_high_ = 4.75, SD = 1.23; *F*_(1,218)_ = 3.88, *p* = 0.05, partial η^2^ = 0.02].

**Figure 4:**
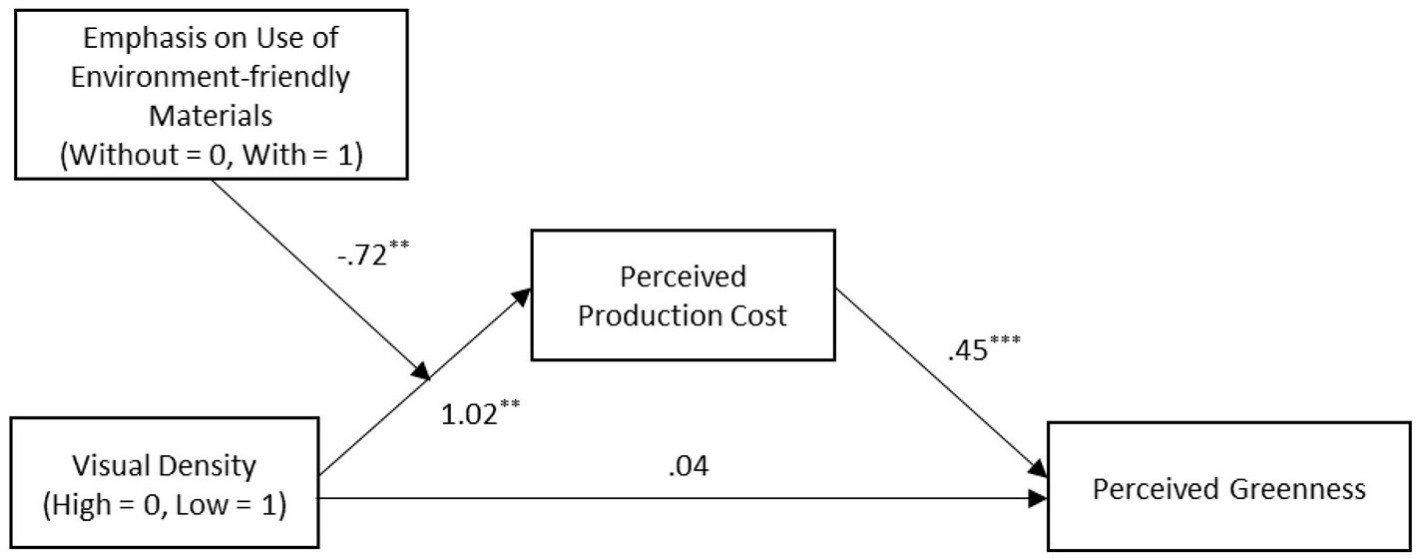
The moderation effect of emphasizing the use of environment-friendly materials. *p <0.1, **p <0.05, and ***p <0.01. Unstandardized coefficients are reported.

To test the moderated mediation, a bootstrapping analysis (PROCESS model 7; Hayes, 2017) with 5000 resamples was conducted (Figure 4). The index of moderated mediation was significant (CI_95%_ [−0.68, −0.07]). The model showed that the interaction between visual design density and emphasis on the use of environment-friendly materials could significantly influence perceived production cost (*b* = −0.72, SE = 0.30, CI_95%_ [−1.34, −0.12]). Perceived production cost significantly influences perceived greenness (*b* = 0.45, SE = 0.05, CI_95%_ [0.35, 0.55]). The indirect effect of perceived production cost was not significant when the use of environment-friendly materials was emphasized (*b* = −0.13, SE = 0.10, CI_95%_ [−0.35, 0.05]), but it was considered significant without the emphasis on environment-friendly materials (*b* = 0.20, SE = 0.11, CI_95%_ [0.01, 0.44]).

### 7.4. Discussion

Experiment 4 supported the fact that emphasis on the use of environment-friendly materials showed the moderation effect of visual density on greenness perception that the negative influence of high visual density diminished when presenting the emphasis. We also found that perceived production cost only showed the mediating effect when participants did not see the emphasis on the use of environment-friendly materials. Taken together, Experiment 4 showed that if participants were reminded about the use of environment-friendly materials, they did not evaluate the dense pattern representing wastage of resources. As a result, they improved their greenness perception of environmentally friendly products with dense patterns. H4 was supported.

## 8. General discussion

Four studies supported that low visual density design can improve the perceived greenness of environmentally friendly products. Study 1 provided initial evidence for this effect that consumers' perceived greenness was higher for environmentally friendly products with low (vs. high) visual density design. Study 2 revealed further the underlying mechanism that this effect was mediated by perceived production cost. Specially, the results on the main effect of visual density design are consistent in the two types of samples from different cultures (one individualist, i.e., USA, and the other collectivist, i.e., China). Study 3 and Study 4 revealed two boundary conditions. Study 3 showed that the effect of visual density design attenuated for consumers with weak holistic thinking tendency. Study 4 showed that the effect of visual density design also attenuated when emphasizing the use of environment-friendly materials.

### 8.1. Theoretical contributions

This study contributes to existing research in three ways. First, it fills the lack of research on the visual characteristics of environmental protection products. Previous studies pointed out the influence of visual factors on green product evaluation (Spack et al., 2012; Pancer et al., 2017; Zhang et al., 2019). However, as mentioned above, these studies mainly focus on the effect of color. In addition to color, other visual factors can also affect consumers' evaluation and purchase intention of products or services. Existing marketing research on how these visual factors other than color affect green consumption behavior is insufficient. New visual characteristics that may affect consumers' attitudes are constantly being developed in recent consumer psychology and visual marketing research (Bellezza et al., 2014; Huang et al., 2019; Su et al., 2019; Xiao et al., 2021). Following such research trends, this study enriches prior research on visual characteristics of environmental protection products by uncovering the effect of visual density.

Second, this study extends the effect of visual density. Visual density is a relatively new visual feature in consumer psychology research (Su et al., 2019). Research on visual density has received very limited discussion so far. In addition to the finding that high visual density improves the difficulty in information processing (Pieters et al., 2010), existing studies also found a metaphorical connection between visual density and psychological emptiness (Su et al., 2019). Other metaphorical meanings of visual density still need further research and exploration. This study demonstrates the impact of visual density on consumers' green product evaluation, which enriches relevant exploration.

Third, the current study advances the field of visual features. We demonstrate that the detrimental impact of high visual density is mitigated when the emphasis is placed on the use of environmentally sustainable materials. It lends credence to the contextual feature of a metaphorical meaning. Previous studies on color pointed out that the metaphorical meaning of visual characteristics will change in different contexts (Elliot and Maier, 2012; Meier et al., 2012). For example, in the competition context, red is associated with aggression, while in the mating context, the red characteristics of the female are associated with sexual attractiveness (Elliot and Maier, 2012; Meier et al., 2012; Pazda et al., 2016). Later, in the research of Zhang et al. (2019), they found that people pay more attention and give more positive rates to blue ads about protecting the sea or green ads about protecting the forest. This means in different situations, the change of the relationship between visual features and objects/concepts affects the metaphorical meaning of visual features. However, previous studies mainly focused on the inherent relationship between visual features and objects/concepts, such as red and blood, blue and sea, green and forest. This study confirms further the fact that direct emphasis on a certain kind of object/concept with a similar conceptual structure is enough to guide consumers to generate new conceptual connections (i.e., high-density design and more use of environment-friendly materials). Further support also extends the situational nature of the psychological meaning of visual features proposed in previous theories.

### 8.2. Practical implications

In practical implications, this study offers a feasible way for marketers to improve consumers' evaluation of environmentally friendly products and instructs marketers to choose the appropriate visual design for these products. For marketers, green marketing makes consumers feel that the products are environmentally friendly. The benefits of green products, such as a higher sense of brand responsibility and image, become more potent when several consumers believe the product is green (Gershoff and Frels, 2015). This study proposes a novel way for visual design, that is, by reducing the visual density of product appearance design so that consumers can improve their evaluation of product greenness.

It is important to promote green consumption for environmental protection (Trivedi et al., 2018; Lu et al., 2021; Chen et al., 2022; Wenting et al., 2022). The fundamental aspect of environmental conservation is how to spread awareness on the idea of green consumption and develop green consumption behaviors (Guo et al., 2020; Chen et al., 2022). Consumers believe that businesses exist to earn money, and using green marketing is only one more way for businesses to achieve this purpose. Apple, which has removed the charger from the iPhone 12, is a fine example. They claimed that this is for environmental aims. However, many consumers believed that Apple's purpose was to reduce costs rather than protect the environment. This study found that the use of low visual density design can make consumers inclined to believe that the product can protect the environment and save resources. Low visual density design may boost consumers' willingness to buy eco-friendly products in the future. Therefore, employing low visual density design may also help customers to understand better the need for businesses to protect the environment and make it easier for them to accept the idea of green consumption.

### 8.3. Limitations

The study mainly identifies the importance of the visual density design of environmentally friendly products. The following restrictions serve as guidelines for future studies. First, consistent with previous studies, this study mainly explores the role of visual density design in product appearance. Previous studies on color found that visual design can alter people's attitudes toward advertising environmental protection. As a result, future research could broaden the application of visual density design. For example, verifying the effect of visual density design in print advertising, environmental protection web page design, environmental protection organization logo design, and other scenarios.

Second, this study mainly explores consumers' evaluation of green products. As mentioned above, making people accept the concept of green consumption and form green consumption habits is significant to environmental protection. Therefore, combined with the carrier of extended visual density design, future research can explore further the impact of visual density design on consumers' future green consumption behavior. For example, when advertisements on environmental protection are designed with low visual density, they may improve consumers' recognition of the concept of green consumption and choose more green products.

Additionally, although we tested the main effect of visual density using the MTurk sample, the mediator and two moderators were tested on university students. Further research could provide support by using different samples.

Moreover, the two moderators proposed the study to target the consumer and business publicity levels in marketing activities. It should be noted that some studies have begun to propose the impact of social environment on consumer choice in recent times. For example, social mobility (Yoon and Kim, 2018), the level of income equality (Blake et al., 2018), gender equality (Blake et al., 2018), and the coronavirus disease (COVID-19) pandemic (Chen et al., 2022). Some studies confirmed that these macro factors may affect consumers' preferences for different visual designs (Batra and Ghoshal, 2017). Therefore, future research can focus further on the interaction between macro social factors and visual density on green consumption.

## Data availability statement

The raw data supporting the conclusions of this article will be made available by the authors, without undue reservation.

## Ethics statement

The studies involving human participants were reviewed and approved by the local legislation and institutional requirements. The patients/participants provided their written informed consent to participate in this study.

## Author contributions

All authors listed have made a substantial, direct, and intellectual contribution to the work and approved it for publication.
