# Supplementary material for: Dense is not green: How visual density influences greenness evaluation on environmentally friendly products
Source: Front Psychol. 2023 Jan 9;13:1035021. doi: 10.3389/fpsyg.2022.1035021 (PMC9869244; doi:10.3389/fpsyg.2022.1035021)

**Appendix A: materials used in experiments**

|  | High visual density | Low visual density |
| --- | --- | --- |
| Experiment 1 | 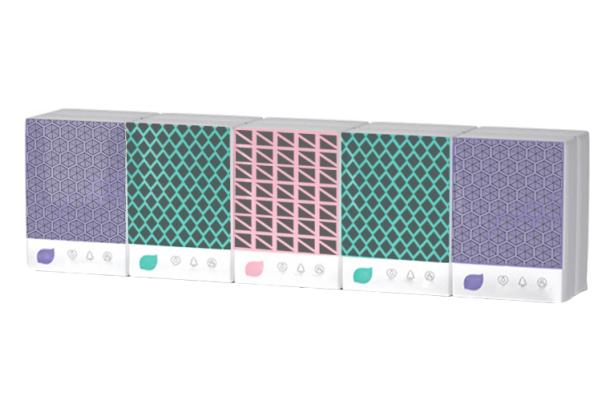 | 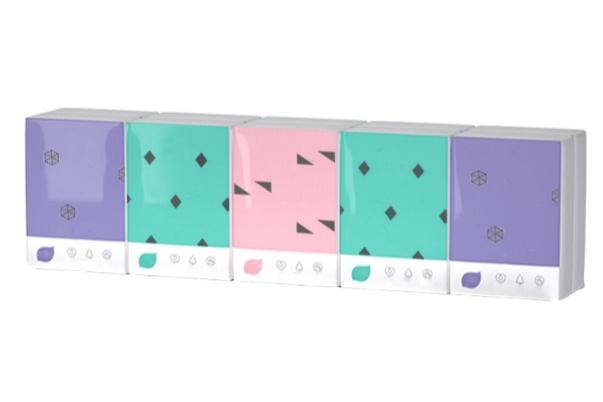 |
| Experiment 2 | 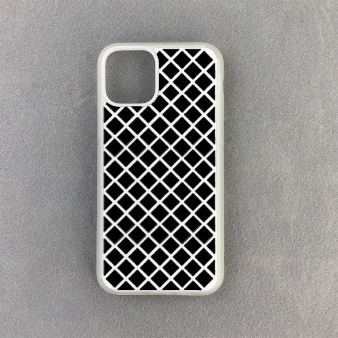 | 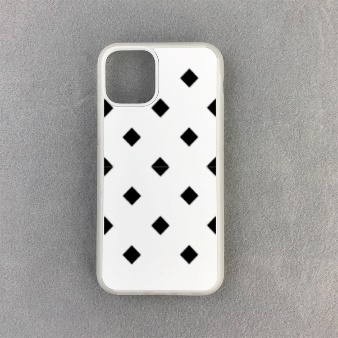 |
| Experiment 3 | 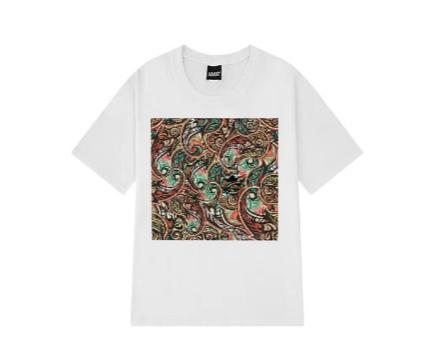 | 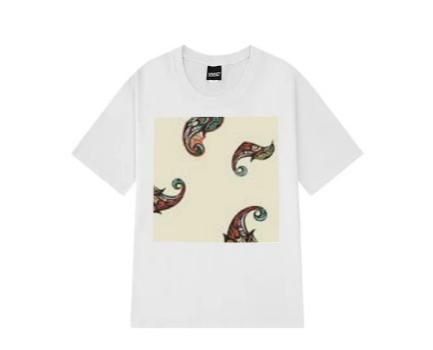 |
| Experiment 4 | 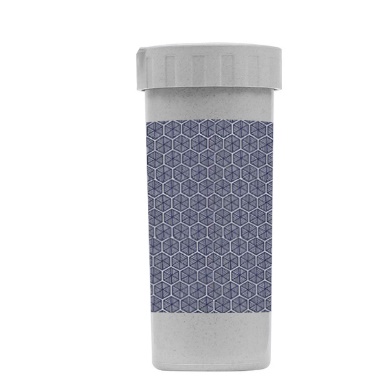 | 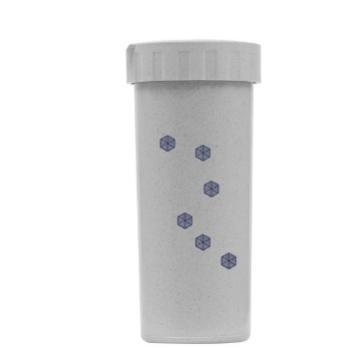 |

**Appendix B**

**Experiment 1**

Thank you for participating in this survey.

Attention please:

1.Please read the following pictures and text carefully and answer according to your first impression and real thoughts.

2.All the questions and answers do not involve the examination of personal knowledge ability.

3.The questionnaire is completely anonymous and the answers you provide are for academic research only.

- **A company has launched a new eco-friendly draw paper which is more friendly to the environment. Imagine that you have noticed the paper in a supermarket, as shown in the picture below.**

Please rate how you feel about the Eco-Friendly Tissue based on the product design above.

- **This tissue is environmentally friendly.**

1 strongly disagree (1)

2 (2)

3 (3)

4 (4)

5 (5)

6 (6)

7 strongly agree (7)

- **This tissue is a good environmental choice**

1 strongly disagree (1)

2 (2)

3 (3)

4 (4)

5 (5)

6 (6)

7 strongly agree (7)

- **gender**

Male (1)

Female (2)

- **age**

________________________________________________________________

- **what is the number in the picture**

________________________________________________________________


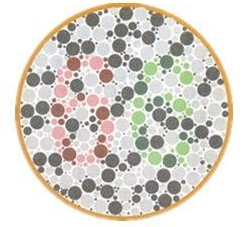


**Experiment 2**

Thank you for participating in this survey.

Attention please:

1.Please read the following pictures and text carefully and answer according to your first impression and real thoughts.

2.All the questions and answers do not involve the examination of personal knowledge ability.

3.The questionnaire is completely anonymous and the answers you provide are for academic research only.

- **A company has launched a phone case which is more friendly to the environment. Imagine that you have noticed the phone case in a supermarket, as shown in the picture below.**

Please rate how you feel about the Eco-Friendly phone case based on the product design above.

- **This phone case is environmentally friendly.**

1 strongly disagree (1)

2 (2)

3 (3)

4 (4)

5 (5)

6 (6)

7 strongly agree (7)

- **This phone case is a good environmental choice**

1 strongly disagree (1)

2 (2)

3 (3)

4 (4)

5 (5)

6 (6)

7 strongly agree (7)

Please rate how you feel about production cost.

- **The merchant saves a lot of resources when making the mobile phone case.**

1 strongly disagree (1)

2 (2)

3 (3)

4 (4)

5 (5)

6 (6)

7 strongly agree (7)

- **The merchant saves a lot of time when making the mobile phone case.**

1 strongly disagree (1)

2 (2)

3 (3)

4 (4)

5 (5)

6 (6)

7 strongly agree (7)

- **The merchant saves a lot of energy when making the mobile phone case.**

1 strongly disagree (1)

2 (2)

3 (3)

4 (4)

5 (5)

6 (6)

7 strongly agree (7)

Please rate your purchase intention.

- **I am willing to buy this product for environmental protection.**

1 strongly disagree (1)

2 (2)

3 (3)

4 (4)

5 (5)

6 (6)

7 strongly agree (7)

- **I am willing to use this product for environmental protection.**

1 strongly disagree (1)

2 (2)

3 (3)

4 (4)

5 (5)

6 (6)

7 strongly agree (7)

- **I am willing to search for relevant information of this product for environmental protection.**

1 strongly disagree (1)

2 (2)

3 (3)

4 (4)

5 (5)

6 (6)

7 strongly agree (7)

- **Please rate your arousal level.**

1= This product is sluggish——— 9=This product is frenzied.

1= This product is calm——— 9=This product is excited.

1= This product is relaxed——— 9=This product is stimulated.

- **gender**

Male (1)

Female (2)

- **age**

________________________________________________________________

- **what is the number in the picture**

________________________________________________________________


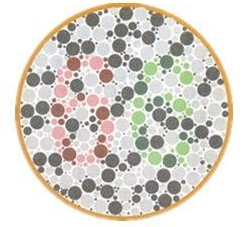


**Experiment 3**

Thank you for participating in this survey.

Attention please:

1.Please read the following pictures and text carefully and answer according to your first impression and real thoughts.

2.All the questions and answers do not involve the examination of personal knowledge ability.

3.The questionnaire is completely anonymous and the answers you provide are for academic research only.

- **A company has launched T-shirt which is more friendly to the environment. Imagine that you have noticed the T-shirt in a supermarket, as shown in the picture below**

Please rate how you feel about the Eco-Friendly T-shirt based on the product design above.

- **This T-shirt is environmentally friendly.**

1 strongly disagree (1)

2 (2)

3 (3)

4 (4)

5 (5)

6 (6)

7 strongly agree (7)

- **This T-shirt is a good environmental choice**

1 strongly disagree (1)

2 (2)

3 (3)

4 (4)

5 (5)

6 (6)

7 strongly agree (7)

- **Even a small change in any element in the universe can lead to substantial alterations in others.**

1 strongly disagree (1)

2 (2)

3 (3)

4 (4)

5 (5)

6 (6)

7 (7)

8 (8)

9 strongly agree (9)

- **Any phenomenon has a numerous results although some of the results are not known.**

1 strongly disagree (1)

2 (2)

3 (3)

4 (4)

5 (5)

6 (6)

7 (7)

8 (8)

9 strongly agree (9)

- **The whole is greater than the sum of its parts.**

1 strongly disagree (1)

2 (2)

3 (3)

4 (4)

5 (5)

6 (6)

7 (7)

8 (8)

9 strongly agree (9)

- **A marker of good architecture is how harmoniously it blends with other buildings around it.**

1 strongly disagree (1)

2 (2)

3 (3)

4 (4)

5 (5)

6 (6)

7 (7)

8 (8)

9 strongly agree (9)

- **Sometimes, the empty space in a painting is just as important as the objects.**

1 strongly disagree (1)

2 (2)

3 (3)

4 (4)

5 (5)

6 (6)

7 (7)

8 (8)

9 strongly agree (9)

- **gender**

Male (1)

Female (2)

- **age**

________________________________________________________________

- **what is the number in the picture**

________________________________________________________________


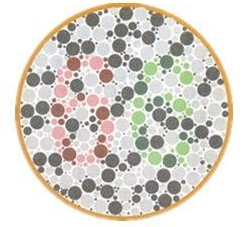


**Experiment 4**

Thank you for participating in this survey.

Attention please:

1.Please read the following pictures and text carefully and answer according to your first impression and real thoughts.

2.All the questions and answers do not involve the examination of personal knowledge ability.

3.The questionnaire is completely anonymous and the answers you provide are for academic research only.

“environmentally-friendly coffee cup” or “environmentally-friendly coffee cup made from coffee grounds and paper”

- **A company has launched a coffee cup which is more friendly to the environment. Imagine that you have noticed the coffee cup in a supermarket, as shown in the picture below.**
- **And Participants were told the product is an “environmentally-friendly coffee cup” or an “environmentally-friendly coffee cup made from coffee grounds and paper”.**

emphasis on the use of environment-friendly materials: environmentally-friendly coffee cup made from coffee grounds and paper

no emphasis on the use of environment-friendly materials: environmentally-friendly coffee cup

Please rate how you feel about the Eco-Friendly T-shirt based on the product design above.

- **This coffee cup is environmentally friendly.**

1 strongly disagree (1)

2 (2)

3 (3)

4 (4)

5 (5)

6 (6)

7 strongly agree (7)

- **This coffee cup is a good environmental choice.**

1 strongly disagree (1)

2 (2)

3 (3)

4 (4)

5 (5)

6 (6)

7 strongly agree (7)

Please rate how you feel about production cost.

- **The merchant saves a lot of resources when making the coffee cup.**

1 strongly disagree (1)

2 (2)

3 (3)

4 (4)

5 (5)

6 (6)

7 strongly agree (7)

- **The merchant saves a lot of time when making the coffee cup.**

1 strongly disagree (1)

2 (2)

3 (3)

4 (4)

5 (5)

6 (6)

7 strongly agree (7)

- **The merchant saves a lot of energy when making the coffee cup**

1 strongly disagree (1)

2 (2)

3 (3)

4 (4)

5 (5)

6 (6)

- **gender**

Male (1)

Female (2)

- **age**

________________________________________________________________

- **what is the number in the picture**

________________________________________________________________


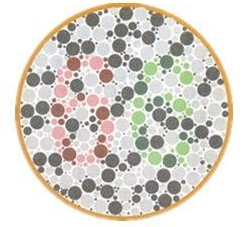

Supplement: Supplementary file 1 [file Table_1.docx]
